# Bortezomib Is Effective in the Treatment of T Lymphoblastic Leukaemia by Inducing DNA Damage, WEE1 Downregulation, and Mitotic Catastrophe

**DOI:** 10.3390/ijms241914646

**Published:** 2023-09-27

**Authors:** Rahman Ud Din, Anan Jiao, Yinxia Qiu, Aarmann Anil Mohinani Mohan, Kei-Ching Yuen, Hoi-Tung Wong, Timothy Ming-Hun Wan, Phoebe On-Yi Wong, Chun-Fung Sin

**Affiliations:** Department of Pathology, Queen Mary Hospital, The University of Hong Kong, 102 Pokfulam Road, Hong Kong, China

**Keywords:** acute lymphoblastic leukemia, proteasome inhibitors, bortezomib, DNA damage, WEE1, mitotic catastrophe

## Abstract

T lymphoblastic leukemia (T-ALL) is an aggressive haematolymphoid malignancy comprising 15% of acute lymphoblastic leukemia (ALL). Although its prognosis has improved with intensive chemotherapy, the relapse/refractory disease still carries a dismal prognosis. Thus, there is an urgent need to develop novel therapy for T-ALL. Bortezomib, a 26S proteasome inhibitor, is licensed to treat plasma cell myeloma and mantle cell lymphoma. Due to its favorable side effect profile, it is a novel agent of research interest in the treatment of ALL. Despite an increasing number of clinical trials of bortezomib in T-ALL, its detailed mechanistic study in terms of DNA damage, cell cycle, and mitotic catastrophe remains elusive. Moreover, WEE1, a protein kinase overexpressed in ALL and involved in cell-cycle regulation, has been known to be a novel therapeutic target in many cancers. But the role of bortezomib in modulating WEE1 expression in ALL still remains elusive. In this study, we demonstrate the therapeutic efficacy of bortezomib on T-ALL primary samples and cell lines. Our findings reveal that bortezomib treatment induces DNA damage and downregulates WEE1, leading to G2-M cell-cycle progression with damaged DNA. This abnormal mitotic entry induced by bortezomib leads to mitotic catastrophe in T-ALL. In conclusion, our findings dissect the mechanism of action of bortezomib and provide further insights into the use of bortezomib to treat T-ALL. Our findings suggest the possibility of novel combination therapy using proteasome inhibitors together with DNA-damaging agents in the future, which may fill the research gaps and unmet clinical needs in treating ALL.

## 1. Introduction

T lymphoblastic leukemia (T-ALL) is a hematological malignancy arising from clonal mutations of immature T-cells, and it comprises 15% of acute lymphoblastic leukemia (ALL) [1]. Despite the improvement in therapeutic outcomes with intensive chemotherapy, the outcome of relapse/refractory disease is still dismal. Early T-cell precursor acute lymphoblastic leukemia (ETP-ALL) is a distinct subtype of T-ALL with worse treatment outcomes compared with other subtypes of T-ALL with limited therapeutic options available [2,3]. Therefore, there is an urgent need to develop novel therapies to improve the treatment outcome of T-ALL.

Previously, cell-cycle pathway dysregulation had been one of the main molecular pathogeneses in ALL, and the use of the CDK4/6 inhibitor played an important role against T-ALL [4,5,6,7]. Bortezomib (formerly known as PS-341), a 26S proteasome inhibitor, was discovered by Dr. Julian Adams and his colleague for inhibiting proteasome activity to treat cancer and inflammatory diseases [8]. Later, bortezomib underwent various clinical trials, and it was then licensed by the Food and Drug Administration (FDA) for treating plasma cell myeloma and relapsed mantle cell lymphoma. Subsequently, bortezomib attracted much attention in research on treating various types of cancer due to its excellent therapeutic efficacy and safety profile [9]. Bortezomib has been shown to be effective in treating relapsed/refractory pediatric ALL, with good therapeutic efficacy and safety profiles when combined with chemotherapy in clinical studies, with no significant increase in the risk of grade 3–4 hematological toxicities compared with chemotherapy alone [10,11,12]. Despite the proven efficacy of bortezomib in treating ALL from clinical studies, its mechanism of action is unclear. A previous study has shown that bortezomib achieves a therapeutic effect in Ph-positive B-ALL via upregulation of FoXO3 [13]. Bortezomib also exerts a therapeutic effect in T-ALL by inducing apoptosis [10]. In Notch1-mutated T-ALL, bortezomib downregulates transcription factor Sp1 and, thus, suppresses Notch1 activation to exhibit a cytotoxic effect [14]. The modulation of the cell cycle by bortezomib was demonstrated in other hematological malignancies and solid tumors [15,16,17,18]. However, it remains uncertain whether bortezomib causes DNA damage, modulates the cell cycle, or impairs mitosis in T-ALL, and its underlying mechanism has yet to be explored.

Furthermore, WEE1 is a protein kinase that inactivates CDK1-bound cyclin B and leads to G2/M arrest and the presence of unrepaired damaged DNA [19]. A recent study found that WEE1 was overexpressed in adult ALL and that inhibiting WEE1 could achieve a therapeutic effect [20]. Overexpression of WEE1 has been shown to be associated with poor prognosis in various solid tumors [21,22,23]. Thus, targeting WEE1 is an emerging novel therapeutic approach in the treatment of ALL. However, the effect of bortezomib on WEE1 expression has not been well studied in T-ALL so far. In this study, we show that bortezomib treatment induces DNA damage, WEE1 downregulation, and G2/M progression with unrepaired DNA, resulting in mitotic catastrophe in T-ALL. Our findings provide mechanistic insights into the role of bortezomib in the treatment of T-ALL and may lead to the development of novel therapeutic strategies for treating T-ALL in the future.

## 2. Results

### 2.1. Bortezomib Induced Cytotoxicity in T-ALL

**Figure 1:**
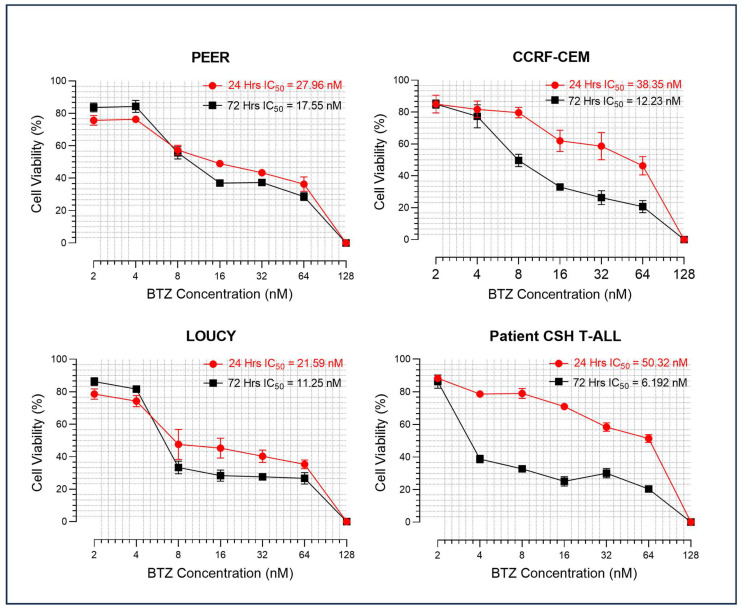
Bortezomib (BTZ) induced cytotoxicity in T Lymphoblastic Leukemia (T-ALL). T-ALL cell lines and primary patient’s sample (Patient CSH T-ALL) were cultured and grown to attain the highest percentage of viability. The cells were then treated for 24 and 72 h with different concentrations of BTZ. Figure corresponds to viability of cells obtained through trypan blue staining after treatment with different concentrations of BTZ. The IC50 values were calculated for both 24 and 72-hour treatments. The experiments were performed independently three times (*n* = 3), and the data were presented as mean ± standard error of mean (SEM). Altogether, the data suggested that BTZ induced cytotoxicity in T-ALL cell lines and primary patient’s sample in dose and time-dependent manner.

T-ALL cell lines were treated with different concentrations of bortezomib for 24 and 72 h, and the cell viability and apoptosis were assessed. The result showed that bortezomib induced cytotoxicity in a dose and time-dependent manner. The IC50 values were calculated both for 24 and 72 h treatment, and the IC50 value at 72 h of treatment was mostly around 10 nM (Figure 1).

**Figure 2:**
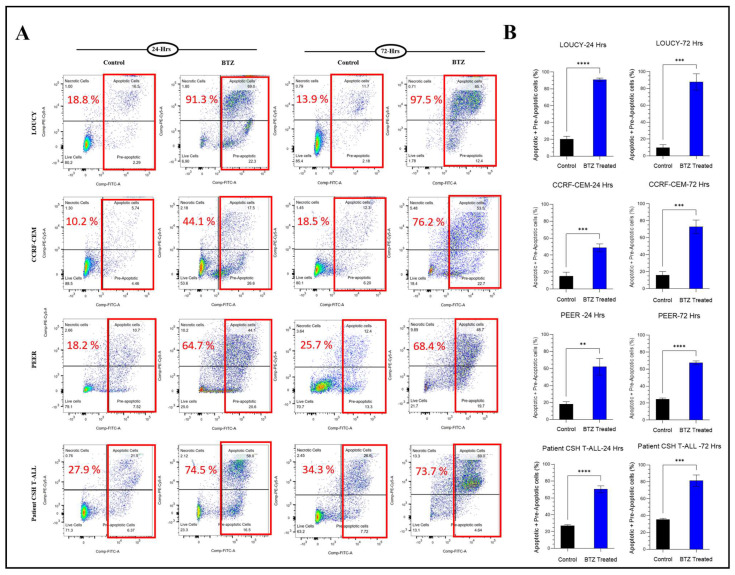
Bortezomib (BTZ) induced apoptosis in T-ALL. T-ALL cell lines (LOUCY, PEER, and CCRF-CEM) and primary patient’s sample (Patient CSH T-ALL) were treated for 24 and 72 h with BTZ (8nM), followed by staining with Annexin V-FITC/PI for flow cytometric analysis of apoptosis. (**A**): Representative flow cytometry plots indicating dramatic increase in percentage of apoptosis after treatment. (**B**): Histograms representing the percentage of apoptosis in vehicle control vs. bortezomib-treated T-ALL cell lines and primary patient’s sample (Patient CSH T-ALL). The percentages were statistically compared, and significant differences are denoted as ****: *p* < 0.0001, ***: *p* < 0.001, **: *p* < 0.01. The experiments were performed independently three times (*n* = 3), and the data were presented as the mean ± standard error of mean (SEM). FITC: Fluorescein Isothiocyanate. PI: Propidium Iodide.

Moreover, the apoptosis induced by bortezomib treatment was assessed by flow cytometric analysis with annexin V/PI double staining. Consistent with our data on cell viability, the percentage of apoptosis was significantly increased in the bortezomib treatment group compared to vehicle control after 24 and 72 h of treatment. The findings showed that bortezomib induced apoptosis in T-ALL (Figure 2A,B). 

### 2.2. Bortezomib Induced Cell-Cycle Changes in T-ALL

**Figure 3:**
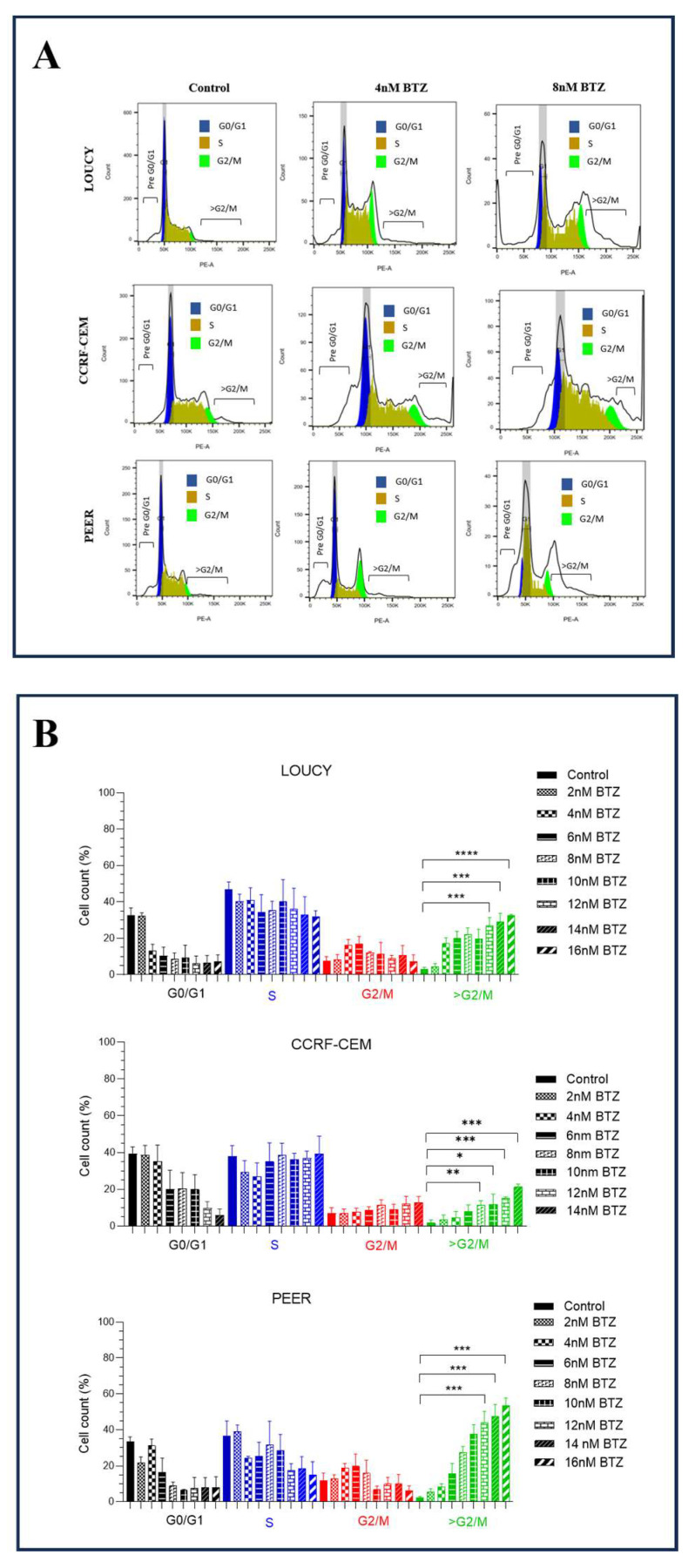
Bortezomib (BTZ) Induced Cell-Cycle Disturbance in T-ALL. T-ALL cell lines were cultured and grown to attain the highest percentage of viability. The cells were then treated for 24 h with different concentrations of BTZ, followed by staining with PI, for flow cytometric analysis of cell cycle. (**A**): Representative cell-cycle flow cytometric plots indicating significant increase in percentage of cells greater than G2/M in bortezomib-treated vs. vehicle control group in T-ALL cells. (**B**): Corresponds to the data of statistical analysis indicating significant increase in percentage of cells greater than G2/M in treated vs. control group in T-ALL cells at different doses of BTZ. The experiments were performed independently three times (*n* = 3), and the data were presented as the mean ± standard error of mean (SEM). Significant differences are denoted as ****: *p* < 0.0001, ***: *p* < 0.001, **: *p* < 0.01, *: *p* < 0.05.

To investigate whether bortezomib inhibited cell proliferation by disrupting the cell cycle, we evaluated the cell-cycle distribution in vehicle control and bortezomib-treated T-ALL cell lines for 24 h using various concentrations. The result showed that bortezomib increased the percentage of cells greater than the G2/M phase in a dose-dependent manner while the percentage of G0/G1 phase cells showed a trend of reduction with the increasing dose of bortezomib treatment, though statistical significance was not reached. (Figure 3A,B). Together, these findings suggested that bortezomib treatment induced cell-cycle progression beyond the G2/M phase in T-ALL. The phenomenon observed would induce chromosomal instability and abnormal mitotic entry.

### 2.3. Bortezomib Induced DNA Damage and Downregulation of WEE1 in T-ALL

**Figure 4:**
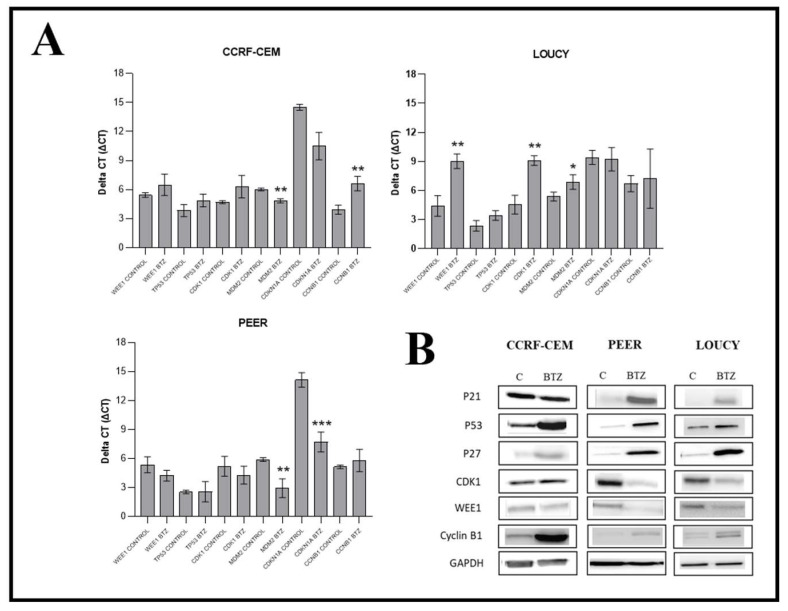
Bortezomib (BTZ) changed mRNA and Protein Expression Profile of Cell-Cycle Regulatory Proteins in T-ALL. T-ALL cell lines were cultured and grown to attain the highest percentage of viability. The cells were then treated for 24 h with different concentrations of BTZ, followed by RNA extraction for real-time quantitative PCR analysis and protein extraction for Western blot analysis. (**A**): Shows the mRNA levels of cell-cycle regulatory genes in T-ALL cell lines. (**B**): Corresponds to Western blot expression profile of cell-cycle regulatory proteins. The result shows that BTZ treatment induced downregulation of WEE1 in all T-ALL cell lines we tested. The experiments were performed independently three times (*n* = 3), and the data were presented as the mean ± standard error of mean (SEM). Significant differences are denoted as ***: *p* < 0.001, **: *p* < 0.01, *: *p* < 0.05.

To further explore the reason for the cell-cycle changes after bortezomib treatment, we assessed the expression level of cell-cycle regulators both at transcriptional and translational levels. Our data show that the mRNA level of CDK1 was significantly reduced in LOUCY, while PEER and CCRF-CEM showed no significant change after 24 h of bortezomib treatment. In line with this, LOUCY showed a significant decrease in the mRNA levels of WEE1 and MDM2, while no significant change was observed in mRNA levels of TP53, CDKN1A, and CCNB1 after 24 h treatment of bortezomib (Figure 4A). Moreover, CCRF-CEM showed a significant decrease in the mRNA level of CCNB1, while there was a significant increase in MDM2, with no change in the levels of WEE1, TP53, and CDKN1A. Similarly, PEER shows a significant increase in the mRNA level of MDM2 and CDKN1A, while no significant change in the level of WEE1, TP53, CDK1, or CCNB1 was observed after 24 h of bortezomib treatment (Figure 4A).

Western blot analysis was performed to further evaluate the changes in cell-cycle regulatory proteins. Our result showed that p21 and p27 were upregulated upon 24 h of bortezomib treatment in all cell lines we tested. The expression of cyclin B1 also increased as well. Moreover, we also observed an increase in p53 levels in all cell lines after bortezomib treatment. In contrast, the expression of CDK1 was reduced upon bortezomib treatment. Interestingly, we find that the expression of WEE1 protein, a regulator of the G2/M checkpoint, was reduced in all cell lines after 24 h of bortezomib treatment, which was in line with our previous data [24] (Figure 4B, Supplementary Figure S1). It has been previously reported that proteasome inhibitors induce DNA double-strand break and block DNA repair in non-small cell lung cancer [25]. The above observations made us hypothesize that bortezomib may cause DNA damage with downregulation of WEE1, resulting in G2/M cell-cycle progression, thus allowing for the cell to enter mitosis with damaged DNA.

**Figure 5:**
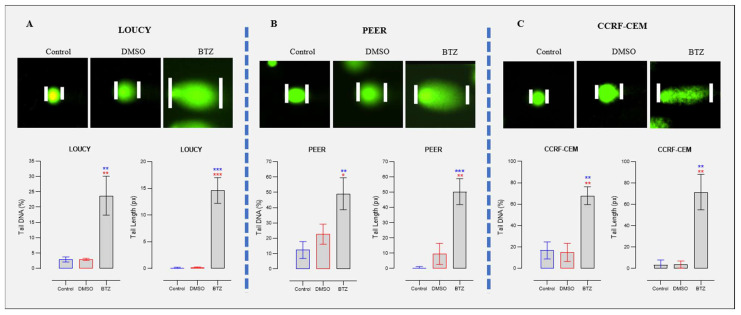
Bortezomib (BTZ) Treatment Causes DNA Damage in T-ALL. T-ALL cells were cultured and grown to attain the highest percentage of viability. The cells were then treated for 24 h with BTZ at various concentrations (LOUCY (8 nM), PEER (8 nM), CCRF-CEM (12 nM)), and DNA damage was assessed using comet assay. (**A**–**C**): Corresponds to Syber gold-stained DNA damage detected by comet assay in T-ALL cells and the statistical analysis showing percentage of DNA damage in treated versus control groups. DMSO was used as a vehicle control (red). Normal control was denoted in blue. The data in BTZ-treated group were individually statistically compared with normal control (blue) and vehicle control (red). The experiments were performed independently three times (*n* = 3), and each time, 50 cells were analyzed for comet analysis from each group. All the images were captured using 20× objective lens. The data were presented as the mean ± standard error of mean (SEM). Significant differences are denoted as ***: *p* < 0.001, **: *p* < 0.01, *: *p* < 0.05.

To verify the hypothesis of bortezomib-induced DNA damage, we assessed the DNA damage by DNA fragmentation assay (Supplementary Figure S4). Our results show the effect of DNA fragmentation in the bortezomib-treated group when compared with the vehicle control group, which suggested the presence of DNA damage induced by bortezomib (Supplementary Figure S4). To further confirm the important observation of DNA damage induced by bortezomib, we conducted a comet assay. Our data showed a significant increase in the comet tail length and comet DNA percentage in the bortezomib-treated group versus vehicle control, which again confirmed the DNA damage induced by bortezomib in all T-ALL cell lines we tested (Figure 5A–C). From the above observations, we concluded that bortezomib induced DNA damage and downregulated WEE1, which allowed the cell to have G2/M cell-cycle progression with damaged DNA. Premature mitotic entry has been shown by various studies to cause mitotic catastrophe and subsequent cell death [26]. 

### 2.4. Bortezomib Cytotoxicity Was Reduced in WEE1 Overexpressing Cells

**Figure 6:**
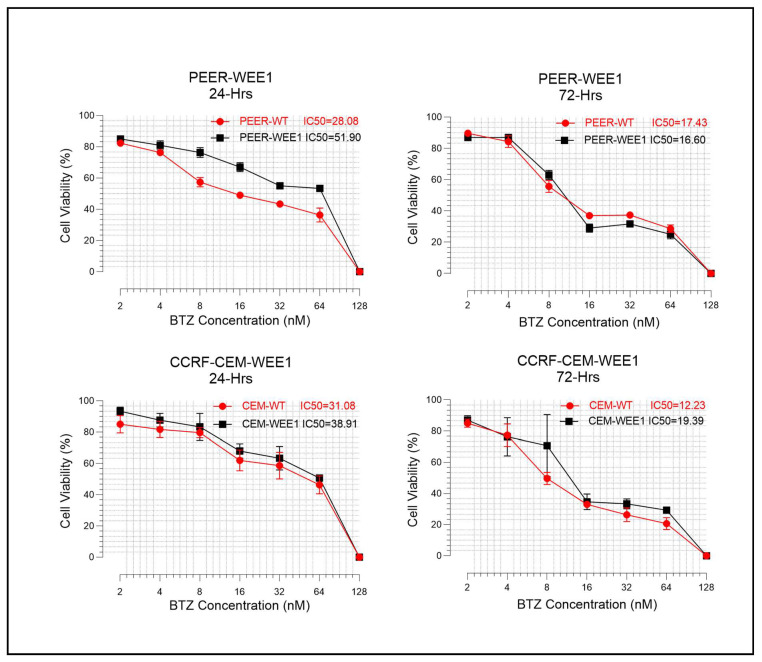
Bortezomib (BTZ) Efficiency Decreases Against WEE1 Overexpressing T-ALL Cells. WEE1 overexpressing cell lines were cultured and then treated with BTZ along with wild-type cell lines for 24 and 72-h. The figure shows a decrease in viability of cells upon treatment with BTZ for 24 and 72 h. The degree of reduction in cell viability was higher in wild-type (red) compared to WEE1-expressing cells (black). Furthermore, the IC50 values for BTZ were calculated and presented in each figure; the result shows reduced IC50 values for wild-type cells compared to WEE1 overexpressing cells both in 24 and 72 h treatment. The experiments were performed independently three times (*n* = 3), and the data were presented as mean ± standard error of mean (SEM).

**Figure 7:**
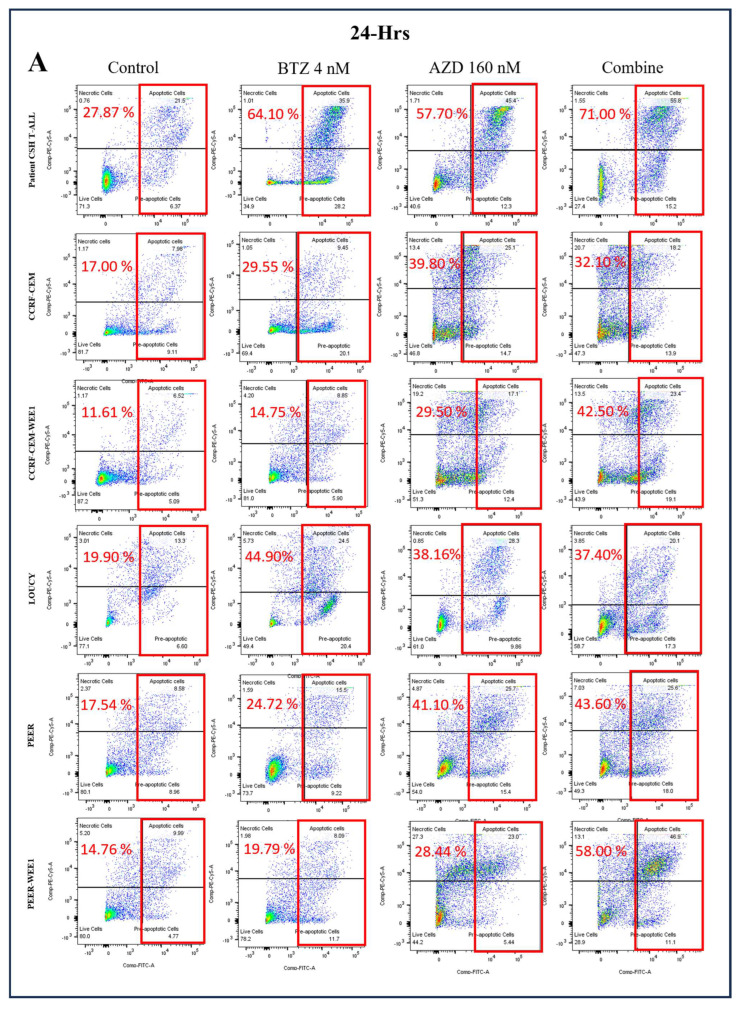

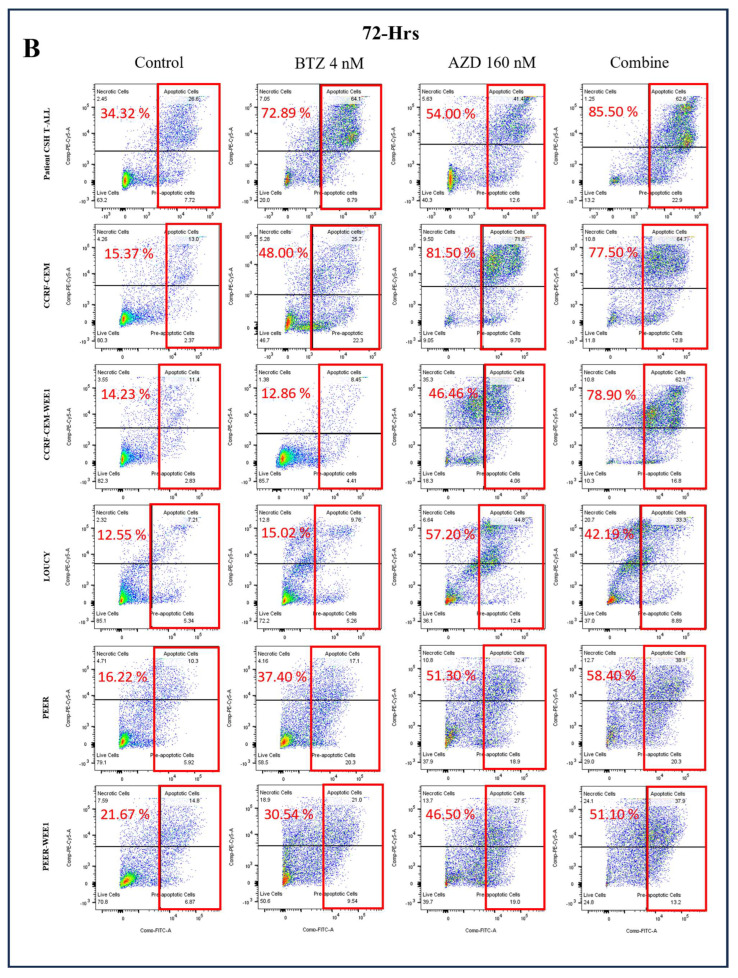

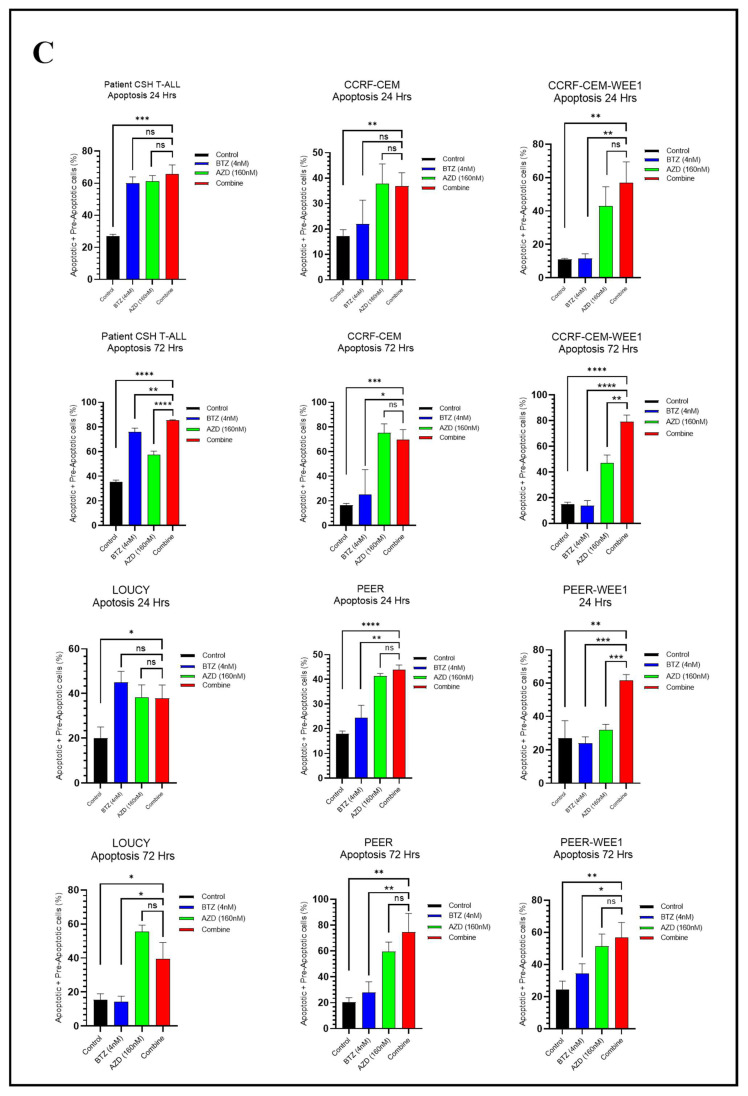
Combined Bortezomib (BTZ) and WEE1 inhibitor (AZD) induce apoptosis in T-ALL Cells. T-ALL cells, including wild-type, WEE1 overexpressing cell lines, and primary patient’s sample (Patient CSH T-ALL), were treated for 24 h and 72 h with BTZ (4 nM) and AZD (160 nM) alone and in combination, followed by staining with Annexin V-FITC/PI for flow cytometric analysis of apoptosis. (**A**): Representative flow cytometry plots after 24 h treatment, indicating dramatic increase in apoptotic plus pre-apoptotic cells after treatment. (**B**): Representative flow cytometry plots after 72 h treatment, indicating dramatic increase in apoptotic plus pre-apoptotic cells after treatment. (**C**): Histograms representing the percentage of pre-apoptotic and apoptotic cells in control, BTZ, AZD, and combined treated T-ALL cells. The percentages were statistically compared, and significant differences are denoted as ****: *p* < 0.0001, ***: *p* < 0.001, **: *p* < 0.01, *: *p* < 0.05. The experiments were performed independently three times (*n* = 3), and the data were presented as the mean ± standard error of mean (SEM). FITC: Fluorescein Isothiocyanate. PI: Propidium Iodide.

In order to figure out the effect of bortezomib on WEE1 overexpressing cell lines, we successfully transfected the WEE1 recombinant plasmid in CCRF-CEM and PEER cell lines. After confirming the successful transfection and overexpression of the WEE1 plasmid (Supplementary Figures S2, S3, and S5), we treated the cells with different concentrations of bortezomib for 24 and 72 h, and the cell viability and apoptosis were assessed. The result showed that the effect of cytotoxicity and apoptosis of bortezomib were hampered in WEE1 overexpressing cells (Figure 6 and Figure 7A,B). We further calculated the IC50 values for both 24 and 72 h treatment, and in line with our previous observation, the IC50 values of bortezomib were higher in WEE1 overexpressing cells compared to wild-type (Figure 6). This observation suggested that WEE1 downregulation was involved in the therapeutic effect of bortezomib treatment in T-ALL.

### 2.5. The Effect of Combine Bortezomib with Adavosertib in Wild-Type and WEE1 Overexpressing T-ALL Cells

To further explore the effect of combined treatment of bortezomib and WEE1 inhibitor, we treated the T-ALL cell lines with bortezomib alone and combined with Adavosertib (AZD), a first-in-class selective WEE1 inhibitor. Our findings suggested that the combined treatment of bortezomib and AZD had an antagonistic effect (CI > 1) after 24 h and 72 h of treatment in wild-type T-ALL cell lines including PEER, CCRF-CEM, and LOUCY (Figure 7A,B and Figure S6 and Supplementary Table S2).

Furthermore, we also treated WEE1 overexpressing cell lines, PEER-WEE1 and CCRF-CEM-WEE1, with bortezomib and AZD alone and in combination for 24 h and 72 h. Our results suggested that the combined treatment of bortezomib and AZD was additive in WEE1 overexpressing cell lines with CI = 1 (Figure 7A,B and Figure S6 and Supplementary Table S2). 

### 2.6. Bortezomib Induced Mitotic Catastrophe in T-ALL

**Figure 8:**
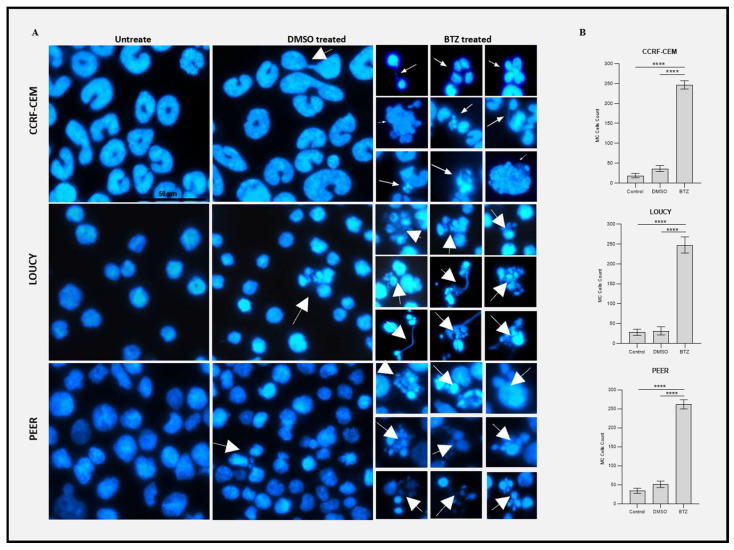
Bortezomib (BTZ) Treatment Induce Cell Death in T-ALL via Mitotic Catastrophe. T-ALL cell lines were cultured and grown to attain the highest percentage of viability. The cells were then treated for 24 h with various concentrations of BTZ (PEER and LOUCY 8 nM, CCRF-CEM 16 nM). The nuclei in each group were stained with DAPI, and images were captured by fluorescence microscope using 20× objective lens. Three hundred cells per group were analyzed, and the number of mitotically defective cells (MC) was counted. (**A**): Corresponds to fluorescence microscopy images of DAPI-stained nuclei in each treatment group. White arrows show cells with mitotic defects. (**B**): Corresponds to the statistical analysis of mitotically defective cells in the treated versus normal control and vehicle groups. DMSO was used as a vehicle control. The experiments were performed independently three times (*n* = 3), and the data were presented as the mean ± standard error of mean (SEM). Significant differences are denoted as ****: *p* < 0.0001. The data suggest that BTZ treatment induces mitotic catastrophe in T-ALL.

To further explore whether bortezomib induces premature mitotic entry and subsequent mitotic catastrophe in T-ALL, we used fluorescence microscopy to study the mitotic disturbance. We divided the cells into three groups, i.e., untreated (normal control), DMSO-treated (vehicle control), and bortezomib-treated group. The majority of the cells in the untreated control and DMSO-treated groups show normal mitotic phases. We observed that the bortezomib-treated group exhibited characteristic nuclear features, including nuclear fracture, nuclear bridge formation, micro-nuclei development, and multiple nuclear lobes formation (Figure 8A,B). These features have been previously reported in abnormal chromosome assembly in metaphase, abnormal chromosome segregation in anaphase, and incomplete karyokinesis exhibiting mitotic catastrophe in cancer cells [27,28,29,30,31]. The number of mitotically defective cells in the bortezomib-treated group was significantly higher compared to normal control and vehicle control groups (Figure 8B). In conclusion, our data showed that bortezomib induced DNA damage and downregulation of WEE1, resulting in premature mitotic entry with damaged DNA, which caused mitotic catastrophe in T-ALL. 

### 2.7. Efficacy of Bortezomib, WEE1 Inhibitor, and Combined Treatment in Primary Patient’s Sample

To further explore the efficacy of bortezomib and WEE1 inhibitor in the primary patient’s sample, we treated the leukemic cells of a patient newly diagnosed with T-ALL with bortezomib for 24 and 72 h. Supplementary Table S1 shows the details of the primary patient’s sample. Bortezomib showed a reduction in cell viability and increased apoptosis after treatment for 24 and 72 h (Figure 1 and Figure 2A,B). The IC50 of bortezomib was well below 10 nM after treatment for 72 h. The sample was also treated with WEE1 inhibitor alone and in combination with bortezomib for 24 h (Figure 7A,B and Figure S6 and Supplementary Table S2). The findings showed a combination index of 1, i.e., additive effect. 

## 3. Discussion

Although the treatment of T-ALL has been improved recently with the introduction of intensive chemotherapy and a risk-adapted treatment regimen, it is still a highly lethal disease, especially in relapse/refractory cases [32]. In our study, we showed that bortezomib downregulated WEE1 with subsequent G2/M cell-cycle progression and DNA damage, resulting in mitotic catastrophe. Despite the proven therapeutic efficacy from clinical studies, the mechanism of action of bortezomib in T-ALL is unclear. The findings shielded the light of important mechanistic insights of bortezomib in treating T-ALL, especially in the aspect of cell-cycle modulation, DNA damage, and mitotic catastrophe. 

Bortezomib was shown to suppress NF-kB pathway and trigger apoptosis [14,33]. Hyperactivation of the NOTCH1 signaling pathway modulates the IKK kinase complex, resulting in the activation of NF-kB pathway [34]. It has been shown that bortezomib induced apoptosis, with or without combination with chemotherapy, on NOTCH1-mutated cell lines or patient’s leukemic cells via modulation of the NF-κB pathway [10,35,36]. However, in vitro studies and preclinical studies to evaluate the effect of bortezomib on NOTCH1 wild-type T-ALL and underlying mechanisms are lacking. In addition, studies on the aspect of DNA damage and mitotic catastrophe of ALL induced by bortezomib are scarce. The findings of our study somehow filled these research gaps. Our study showed that a single treatment with bortezomib was active against all T-ALL cell lines, including LOUCY, which has an ETP-ALL phenotype with NOTCH1 wild-type status [37]. The IC50 of these cell lines was comparable to that of plasma cell myeloma and mantle cell lymphoma (both around 10 nM) [38,39]. The findings provide insights for developing a novel therapeutic approach to T-ALL. 

In our study, we demonstrated evidence of DNA damage upon bortezomib treatment in T-ALL. It was proposed that the DNA damage-repairing response is regulated by the ubiquitin–proteasome system [40]. A previous study showed that bortezomib induced DNA damage via interfering DNA repairing response in neuroendocrine tumors, and the effect of DNA damage was synergized with DNA damaging agent [41]. Similar effects of synergism were observed when combining bortezomib with chemotherapeutic agents or irradiation in solid tumors [25,42]. However, no more studies have shown the effect of DNA damage and its underlying mechanism in T-ALL upon bortezomib treatment. Although our study provided insightful evidence of DNA damage induced by bortezomib in T-ALL, further studies are needed to elucidate the mechanism of DNA damage induced by bortezomib in T-ALL.

Furthermore, we observed that the protein level of p53 was increased upon the treatment of bortezomib without significant changes at the mRNA level. Although MDM2 is a negative regulator of p53 via transcriptional and post-translational mechanisms [43], we did not observe a consistent trend of reduction in the MDM2 mRNA level. The findings suggested that bortezomib stabilized p53 protein and prevented its degradation. Bortezomib has been shown to prevent proteosome-mediated degradation of p53 and promote apoptosis in breast cancer cells. Moreover, the increase in p53 expression was a direct result of DNA damage observed in bortezomib treatment [44]. However, the effect of apoptosis could be p53-independent since apoptosis was still observed in p53-null breast cancer cells [45]. Upregulation of p53 was also observed in mantle cell lymphoma after treatment with bortezomib. Moreover, an increase in NOXA expression was noted and promoted the release of BAK/BIM with subsequent events of apoptosis in p53-null mantle cell lymphoma cell lines [46]. However, it is uncertain whether bortezomib can induce p53-independent apoptosis in T-ALL. Further studies are required to investigate the role of p53 in bortezomib-induced apoptosis in T-ALL, especially in TP53-mutated ALL. The presence of TP53 mutations carries a poor prognosis, and this mutation commonly presents in relapsed ALL [47]. 

Previous studies showed that bortezomib induced G2/M cell-cycle arrest in neuroblastoma, CD30-positive anaplastic large cell lymphoma, and mantle cell lymphoma [16,48,49]. Furthermore, G2/M cell-cycle arrest associated with aberrant mitosis or mitotic catastrophe was observed in B-cell lymphoma upon bortezomib treatment [50]. The effect on the cell-cycle modulation by bortezomib had not been extensively studied in T-ALL. Bortezomib has been shown to induce G2/M cell-cycle arrest in ALL with KMT2A-rearrangement via upregulation of p27 by increasing CDKN1B transcription [51]. However, studies to evaluate the phenomenon of mitotic catastrophe and the underlying mechanism in T-ALL upon bortezomib treatment are scarce. Here, our study showed that bortezomib did not induce G2/M cell-cycle arrest, but the progression of the cell cycle beyond G2/M was observed, resulting in a mitotic catastrophe. The findings suggested that there was the presence of G2/M cell-cycle progression with damaged DNA leading to mitotic catastrophe. 

Moreover, we observed WEE1 downregulation upon bortezomib treatment. WEE1 kinase is a G2 m cell-cycle regulator and is overexpressed in various cancers, including ALL. This study showed that ALL with overexpression of WEE1 conferred unfavorable prognosis [20]. Bortezomib reduced the WEE1 expression of B-cell lymphoma cell lines [50], while another study found that bortezomib increased the expression level of WEE1 in vascular endothelial cells [32]. L. Liang et al. failed to show the downregulation of WEE1 by bortezomib in plasma cell myeloma [52]. However, the role of bortezomib in the modulation of WEE1 expression in ALL has not been studied so far. Thus, the results from our study provided good evidence of the downregulation of WEE1 by bortezomib in T-ALL. The protein p53 is the main mediator of the G0/G1 cell-cycle checkpoint in the presence of DNA damage. Leukaemic cells harboring TP53 mutation would result in failure of the G0/G1 checkpoint. WEE1 kinase will halt the cell-cycle progression at the G2 checkpoint [19]. Thus, the downregulation of WEE1 in T-ALL induced by bortezomib would promote mitotic entry with damaged DNA, thus causing a mitotic catastrophe. The overexpression of WEE1 attenuated the therapeutic effect of bortezomib, which indicated that WEE1 downregulation partly contributed to the therapeutic effect of bortezomib. The inhibition of WEE1 could induce cell death via a p53-independent mechanism by modulating the G2 cell-cycle checkpoint, and, therefore, it is an effective treatment strategy for TP53-mutated ALL. Indeed, apoptosis was observed in TP53-mutated leukemic cells via WEE1 inhibition. Combined WEE1 inhibitor (MK1775) and conventional chemotherapy had a synergistic effect in TP53 mutated acute myeloid leukemia [53]. Moreover, this study showed that the synergism of the WEE1 inhibitor (AZD-1775) and clofarabine was due to the potentiation of DNA damage [20]. A recent study also showed that combined bortezomib and WEE1 inhibitor (MK1775) achieved a synergistic effect in multiple myeloma and depleting stem-like cells in multiple myeloma [52]. However, our results did not show a synergistic effect of combined bortezomib and WEE1 inhibitor in both wild-type and WEE1-overexpressing cells, although the combination index in WEE1-overexpressing cells was smaller. We are not sure about the reason behind the antagonism observed in WEE1 wild-type cell lines. However, since WEE1 downregulation at least contributed partly to the therapeutic effect of bortezomib in ALL, the WEE1 inhibition in ALL cells with low-level expression of WEE1 may greatly diminish the effect of WEE1 downregulation by bortezomib and, hence, jeopardize the therapeutic effect of bortezomib. Further studies would be warranted for studying the efficacy of combination with other genotoxic agents, which would potentiate the DNA damage. Further studies are also needed to elucidate the mechanism of WEE1 downregulation in T-ALL upon bortezomib treatment. 

Our study shows the downregulation of CDK 1 and upregulation of cyclin B1 in T-ALL cell lines upon bortezomib treatment. This study showed that bortezomib had a similar effect on CDK1 and cyclin B1 in non-small cell lung carcinoma cell lines [18]. Another study showed that bortezomib increased cyclin B levels in NK/T-cell lymphoma. Moreover, despite the total CDK1 being reduced, the CDK1/Cyclin B activity was increased by increasing phosphorylation at CDK1 upon bortezomib treatment, thus resulting in a mitotic catastrophe [54]. In our study, the observation of upregulation of cyclin B explained the phenomenon of G2/M cell-cycle progression. However, the exact changes in CDK1/Cyclin B activity upon bortezomib treatment in T-ALL, besides the protein expression level, need further investigation.

Our study showed that the expression of p21 and p27 increased after treatment of bortezomib. Previous work has demonstrated that bortezomib stabilizes p27, p53, and p21 protein with G2/M arrest [17,55,56,57]. In the presence of DNA damage, p21 is responsible for inhibiting the cycling B-CDK1 complex, resulting in G2 cell-cycle arrest [58,59]. P27 is also responsible for inhibiting CDK1 and subsequently leads to G2/M cell-cycle arrest [60]. Therefore, the increase in levels of p21 and p27 may cause G2 cell-cycle arrest. However, we failed to observe G2/M cell-cycle arrest but cell-cycle progression beyond G2/M instead. It is uncertain how the effect of G2/M cell-cycle progression via WEE1 inhibition interacts with the effect of elevated p21 and p27 upon bortezomib treatment. Further mechanistic study on cell-cycle modulation by bortezomib is needed.

In conclusion, our study showed DNA damage, WEE1 downregulation, and mitotic catastrophe induced by bortezomib treatment in T-ALL. This provides a mechanistic exploration of bortezomib action in T-ALL, especially NOTCH1 wild-type T-ALL. We also showed that bortezomib acts through a novel therapeutic target in the treatment of ALL, WEE1 kinase. Further studies are needed to evaluate the mechanism of WEE1 downregulation. Also, further studies need to explore optimal combination therapeutic strategies, especially combined with other DNA-damaging agents. Finally, our data provide opportunities for further research about the use of proteasome inhibitors to treat T-ALL and improve its prognosis.

## 4. Materials and Methods

### 4.1. Drugs, Cytokines, and Antibodies

Bortezomib (BTZ) was purchased from Merck. For the in vitro experiments, a 10 mM stock solution was prepared by dissolving it in dimethyl sulfoxide (DMSO) purchased from Sigma-Aldrich. The concentration of DMSO in the culture was kept from 0.1 to 0.2%. The stock solution was stored at −20 °C until use. The antibodies for the immunoblots, including GAPDH (14C10), P21^waf1/cip1^ (12D1), P53, P27^kip1^ (D69C12), CDK1 (POH1), CYCLIN B1 (D5C10), and WEE1 (D10D2), were purchased from Cell Signaling (Beverly, MA, USA), while Anti-DDK(FLAG) (TA50011-100) was purchased from OriGene Technologies, Inc. (Rockville, MD 20850, USA).

Five cytokines—IL-3, IL-6, SCF, G-CSF, and Flt3-L—were purchased from PEPRO Tech (Rocky Hill, NJ, USA).

### 4.2. Cell Lines and Cell Culture

The human T-ALL cell lines, namely, LOUCY, PEER, and CCRF-CEM were purchased from Deutsche Sammlung von Mikroorganismen and Zellkulturen GmbH (DSMZ) and authenticated. They were cultured in RPMI-1640 (Invitrogen, Waltham, MA, USA) medium supplemented with 10–20% bovine serum (Hyclone, Logan, UT, USA) and 1% penicillin (100 U/mL)/streptomycin (100 ug/mL) (Gibco, Billings, MT, USA). All cultures were maintained at 37 °C in a humidifier atmosphere containing 5% CO_2_.

### 4.3. Primary Patient Sample

A primary patient sample with T-ALL was obtained from bone marrow aspiration of the patient with newly diagnosed T-ALL after obtaining informed consent. The experiment was approved by the Institutional Review Board of HKU/HA HKW (Protocol Number: UW19-809). The marrow blood mononuclear cells (MBMC) were extracted using Ficoll Paque Plus (GE17-1440-02) purchased from MERCK (Darmstadt, Germany). The MBMC were cultured in IMDM + GlutaMAX™ (Gibco Catalog number: 31980030) medium supplemented with 5% fetal bovine serum (FBS), 1% Penicillin-Streptomycin (10,000 U/mL Catalog number: 15140122), and five cytokines (20 nanomole IL-3, 20 nanomole IL-6, 100 nanomole SCF, 20 nanomole G-CSF and 100 nanomole Flt3-L). 

### 4.4. Flow Cytometric Analysis of Apoptosis

Cells were seeded in the 24-well plates at 0.75 × 10^6^ cells/mL with vehicle control and various doses of bortezomib for a duration of 24 h and 72 h. The cells obtained from the treatment were washed with ice-cold phosphate-buffered saline (PBS) after trypsinization and resuspended in the Annexin V binding buffer containing 10 mM Hepes/NaOH (pH 7.4), 140 mM NaCl, and 2.5 mM CaCl_2_. These solutions were mixed to make the total density of the solution equal to 0.75 × 10^6^ cells/mL. Then, 80 μL of the cell solution was mixed with 10 μL of Annexin V FITC (Pharmingen, San Diego, CA, USA), 10 μL of propidium iodide (50 μg /mL in PBS (pH 7.4), and 0.2 μm sterile-filtered). The frequency of apoptotic cells (Annexin V/PI positive cells) was determined by using a minimum of 10,000 events using the FACS Fortress flow cytometer (Becton Dickinson, Lincoln Park, NU, USA). The data of the flow cytometric study were analyzed by using FlowJo_v10.8.1.

### 4.5. Flow Cytometric Analysis of Cell Cycle

Cells were seeded at a density of 1 × 10^6^ cells/well in a 24-well plate treated with vehicle control and various doses of bortezomib, then harvested after 24 h with washing by PBS before being fixed and permeabilized with 2% PFA at 4 °C overnight. The cells were incubated with RNase and propidium iodide. The data were acquired with a FACS Fortressa flow cytometer (Becton Dickinson, Lincoln Park, NJ, USA). FlowJo_v10.8.1 software was used to calculate the proportion of cells in different phases. 

### 4.6. Trypan Blue Staining Assay

The 0.4% Trypan Blue staining solution assay (Thermo Fisher-Scientific, Waltham, USA) can differentiate between live and dead cells by the dye exclusion test. It is an impermeable dye to viable cell membranes, which freely permeates into apoptotic cells. For this procedure, a 1:2 dilution of cell culture was mixed with 0.4% trypan blue stain (10 μL of cell culture was added to 10 μL of trypan blue). A total of 10 μL of the mixture was then loaded onto a TC20 counting slide, which was then inserted into the TC20 automated cell counter (Bio-Rad Inc., Hercules, California), where it was automatically counted.

Viability (%) = viable cell count / total cell count × 100

The viability of the vehicle control and bortezomib-treated cell culture was analyzed in this study.

### 4.7. Immunoblot Analysis

After treatment of the leukemia cells with vehicle control and bortezomib for 24 h, the cells were lysed with RIPA buffer, and the cell lysate samples (50 μg/lane) were separated by sodium dodecyl sulfate-polyacrylamide gel electrophoresis (SDS-PAGE) on 8–12% gels. After separation of proteins, they were transferred to a polyvinylidene difluoride (PVDF) membrane and subsequently blocked with 5% (*v/v*) non-fat dried milk prepared in Tris-buffered saline containing 0.1% tween-20 and incubated with corresponding antibodies at 1:1000 and secondary antibodies at 1:1000. The immunoreactive protein bands were visualized using Alliance Q9 Advanced Chemiluminescence Imager (Uvitec, Cambridge, UK). The expression of p21, p53, p27, CDK1, cyclin B1, and WEE1 were studied via Western blot.

### 4.8. Quantitative RT-PCR Study

Quantitative RT-PCR was performed on cells treated with vehicle control and bortezomib for 24 h. Total RNA was extracted and purified using a mirVana miRNA isolation kit (Thermofisher Scientific, Wilmington, DE, USA). The concentration and quality were measured using nanodrop ND1000 (Thermofisher Scientific, DE, USA) by spectrophotometric determination. A criterion using the 260/280 ratio being over 1.8 was used to determine whether the RNA quality was pure enough to continue to reverse transcription. The RNA was reversed transcribed to cDNA using the High-Capacity cDNA Reverse Transcription Kit according to the manufacturer’s instructions. Primers were designed (Table 1), and qPCR was conducted. The conditions were set to pre-denaturation at 95 °C for 25 s, 40 cycles of amplification with denaturation at 95 °C for 15 s, annealing at 60 °C for 60 s, and extension at 72 °C for 30 s. GAPDH was used as a housekeeping gene to normalize the data.

### 4.9. WEE1 Plasmid and WEE1 Inhibitor

For generating WEE1 overexpressing cell lines, WEE1 (NM_003390) Human Tagged ORF Clone (RC209760), purchased from OriGENE (Rockville, MD 20850, USA), was transfected to T-ALL cell lines using Lipofectamine 3000 Transfection Reagent (ThermoFisher) according to the instruction of package insert. WEE1 inhibitor, Adavosertib (Synonyms: AZD1775; MK-1775), was purchased from MCE (Hong Kong).

### 4.10. Agarose DNA Gel Electrophoresis

Cells were seeded in a 6-well plate at a density of 1.5 × 10^6^ per mL. A total of 4.5 × 10^6^ cells were seeded per well and treated with different bortezomib concentrations for 24 h. After treatment, the cells were harvested, and genomic DNA was extracted according to the package insert of DNeasy Blood and Tissue Kit QIAGEN (Cat. No. 69504). The recovered DNA concentration was measured using Nanodrop. The DNA fragmentation was analyzed by 1% Agarose Gel electrophoresis using a Bio-Rad system (Voltage 180, Time 1:15 h). Gel was visualized using Gel-Doc XR plus system (Bio-Rad) and documented.

### 4.11. Comet Assay for Measuring DNA Damage

CometAssay^®^ Reagent Kit for a Single Cell Gel Electrophoresis Assay Catalog # 4250-050-K was purchased from R&D systems, and the reagents were made according to the manufacturer’s instructions. The alkaline comet assay was performed on cells treated with vehicle control and bortezomib after treatment for 24 h, according to the manufacturer’s instructions. The comet images were taken using a fluorescence microscope (Nikon Live Cell Imaging System). A total of 50 cells per treatment were analyzed, the comet tail length and tail percent DNA were calculated, and the mean value was determined. The images were processed by the automatic comet assay software CometScore Pro 2.0 (2012 TriTek Corp). 

### 4.12. Mitotic Study

The mitotic disturbance induced by bortezomib treatment was studied in T-ALL cell lines after treatment with vehicle control and bortezomib for 24 h by using the previously published protocol with few modifications [61]. The cells were seeded in a density of 0.3 × 10^6^ cells/mL (CCRF-CEM) or 1 × 10^6^ cells/mL (PEER and LOUCY) and treated for 24 h along with the vehicle control. For PEER and LOUCY, the plates were coated with Poly-L-Lysine (0.1% *w/v*, Catalog No. P 8920, SIGMA-ALDRICH CO) prior to seeding the cells. The images of DAPI-stained nuclei were taken using a fluorescence microscope (Nikon Live Cell Imaging System) and were processed using ImageJ software 1.53t. A total of 300 cells from each group were analyzed, and the number of nuclei exhibiting the mitotic catastrophe was counted. 

### 4.13. Statistical Analysis

All the experiments were performed three times independently (*n* = 3). The data are expressed as mean ± standard error of the mean (S.E.M.). The difference in data among groups was calculated by one-way analysis of variance (ANOVA) and post hoc Tukey’s test as appropriate. The difference between groups was determined by the Student’s *t*-test. All statistical analyses were performed using GraphPad Prism 9 (GraphPad Software Inc., La Jolla, CA, USA). A *p*-value of lower than 0.05 was taken as statistically significant. Significant differences are denoted as ***: *p* < 0.001, **: *p* < 0.01, *: *p* < 0.05.

## Figures and Tables

**Table 1:** Forward and Reverse Primers Used in RT-qPCR.

Gene Name	Forward Sequence	Reverse Sequence
GAPDH	GTCTCCTCTGACTTCAACAGCG	ACCACCCTGTTGCTGTAGCCAA
CDK1	GGAAACCAGGAAGCCTAGCATC	GGATGATTCAGTGCCATTTTGCC
WEE1	GATGTGCGACAGACTCCTCAAG	CTGGCTTCCATGTCTTCACCAC
TP53	CCTCAGCATCTTATCCGAGTGG	TGGATGGTGGTACAGTCAGAGC
CDKN1A	AGGTGGACCTGGAGACTCTCAG	TCCTCTTGGAGAAGATCAGCCG
CCNB1	GACCTGTGTCAGGCTTTCTCTG	GGTATTTTGGTCTGACTGCTTGC
MDM2	TGTTTGGCGTGCCAAGCTTCTC	CACAGATGTACCTGAGTCCGATG

## Data Availability

The data used to support the findings of this study are included within the article.

## Supplementary Materials

The following supporting information can be downloaded at https://www.mdpi.com/article/10.3390/ijms241914646/s1.
