# Supplementary material for: Bortezomib Is Effective in the Treatment of T Lymphoblastic Leukaemia by Inducing DNA Damage, WEE1 Downregulation, and Mitotic Catastrophe
Source: Int J Mol Sci. 2023 Sep 27;24(19):14646. doi: 10.3390/ijms241914646 (PMC10572992; doi:10.3390/ijms241914646)
Supplement: Supplementary file 1 [file ijms-24-14646-s001.zip › ijms-2590557-supplementary.pdf]

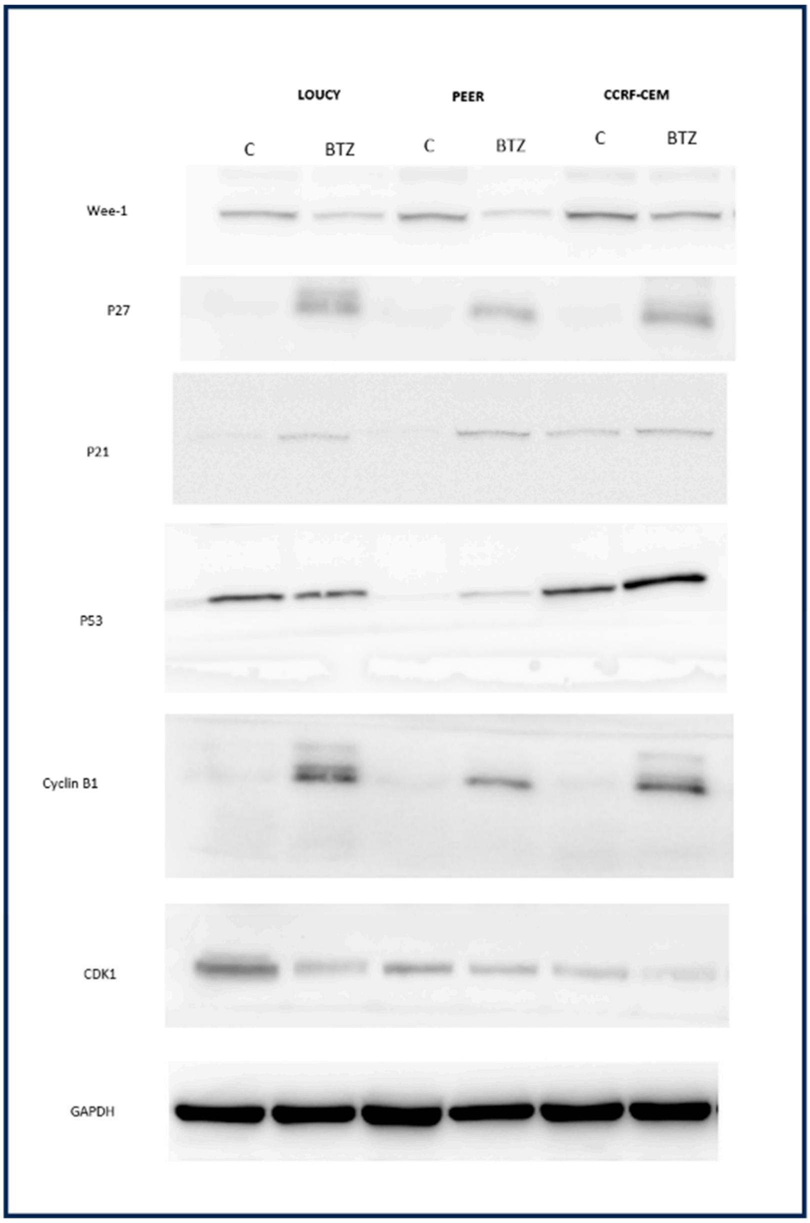

Figure S1. Uncut repeated western blot of Figure 4B.

To verify and obtain the trend of protein expression upon BTZ treatment, we repeated the western blotting experiment. The figure corresponds to the uncut repeated western blot of figure 4B.

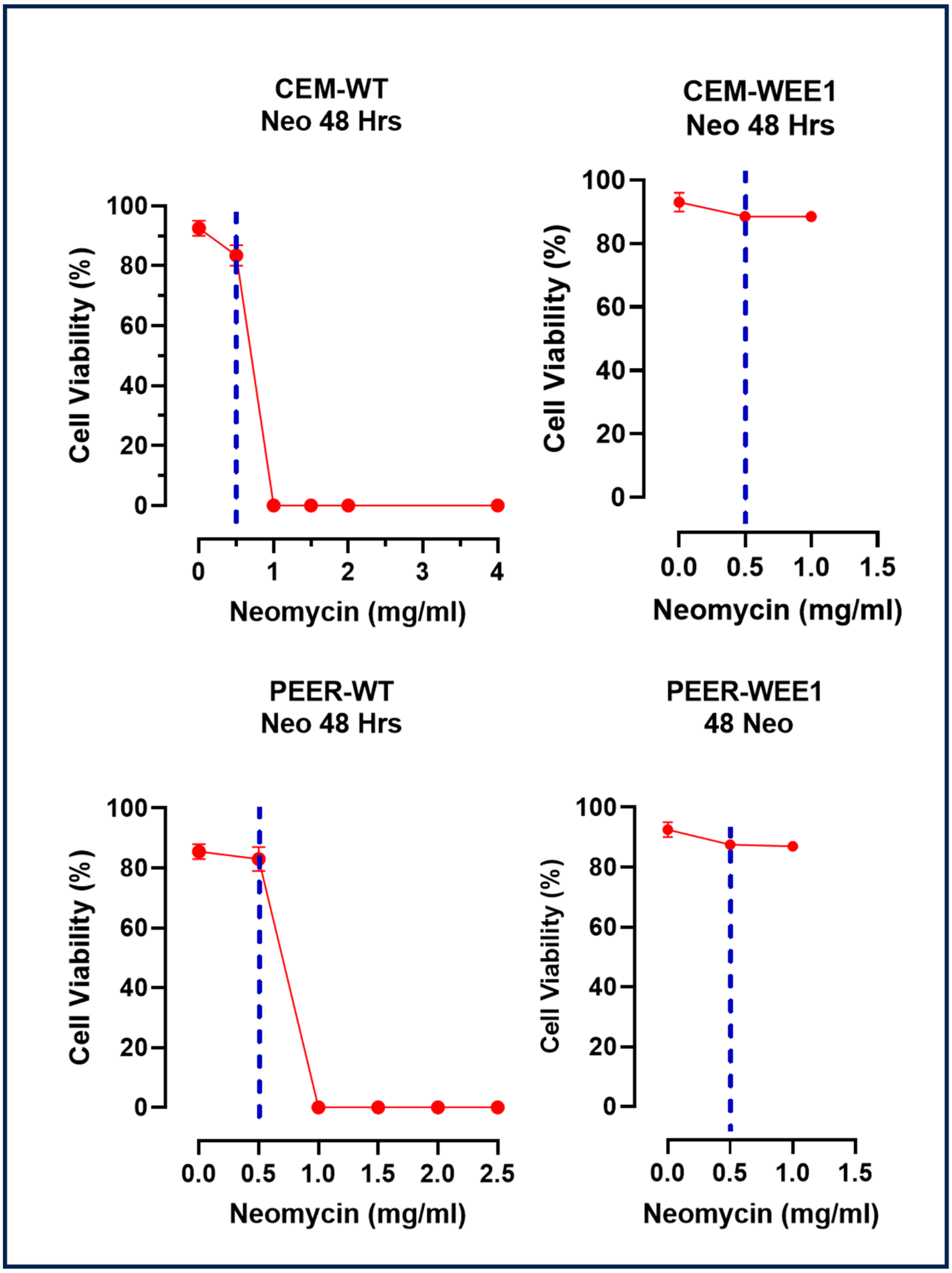

Figure S2. Antibiotics Selection of the WEE1 Plasmid Transfected Cell Lines.

PEER and CCRF-CEM cell lines were transfected with WEE1 plasmid having Neomycin resistance and DDK tag. The transfected cells were collected after culturing the cells in 1 mg/ml of neomycin for 48-hours. The wild type cells do not show any resistance to neomycin while the transfected cells were resistant to neomycin after 48-hours of exposure. The cells were collected and used for further experiments.

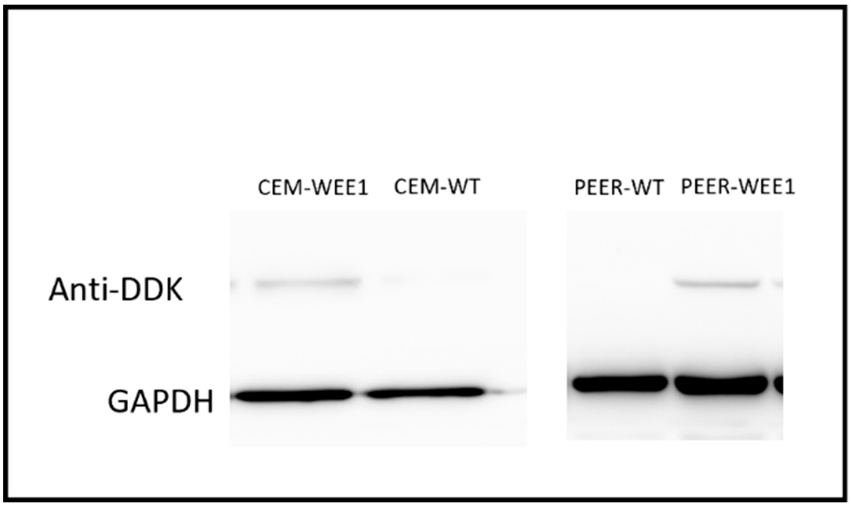

Figure S3. Western Blot Confirmation of WEE1 Plasmid Transfection.

Both the cell lines PEER and CCRF-CEM were transfected with WEE1 recombinant plasmid containing DDK tag and neomycin resistance gene. After transfection, we collected the neomycin resistant cells and performed western blotting using anti-DDK antibody to double confirm the successful transfection of WEE1 plasmid. Our result shows successful transfection and expression of the WEE1 plasmid.

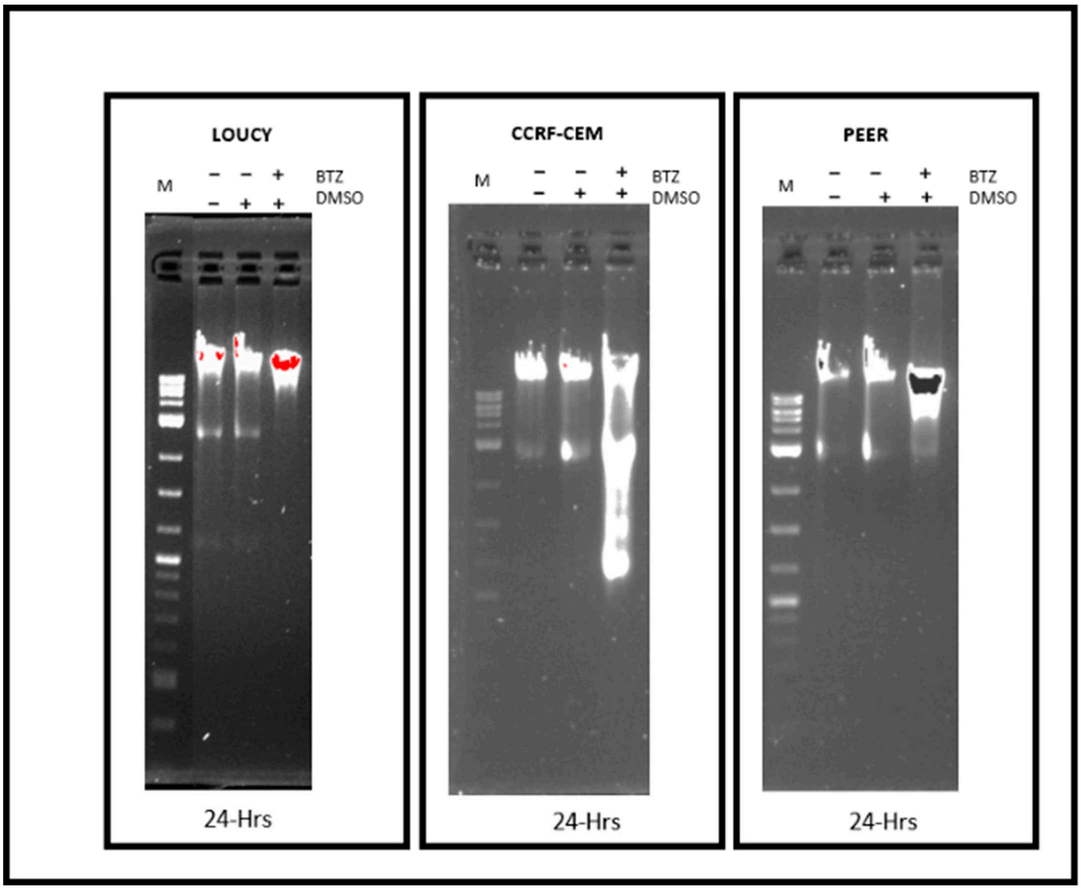

Figure S4. BTZ Treatment Induces DNA Fragmentation in T-ALL Cells.

T-ALL cell lines were grown and treated with BTZ (8nM) and DMSO (vehicle control) for 24-hours. Cells were collected and DNA was extracted followed by agarose gel electrophoresis. The results showed that BTZ treatment induced DNA fragmentation in the treated group compared to controls in LOUCY, CCRF-CEM and PEER cell lines.

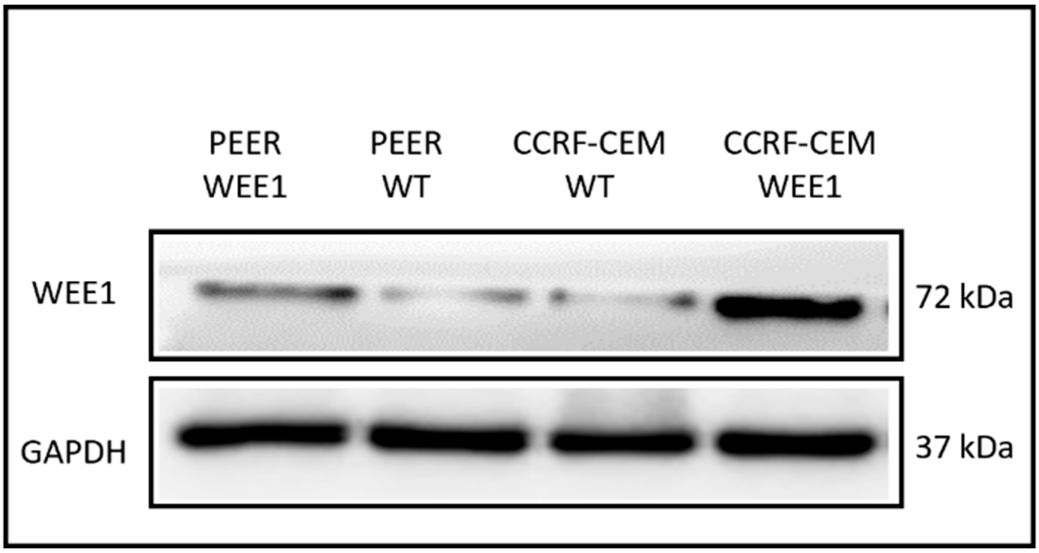

Figure S5. Successful Over Expression of WEE1 Protein in WEE1 Overexpressing Cell Lines.

Figure shows the western blot, using anti-WEE1 antibody, confirming the over expression of WEE1 in WEE1 overexpressing cell lines (PEER WEE1, CCRF-CEM WEE1) compared to wild type (PEER WT, CCRF-CEM WT).

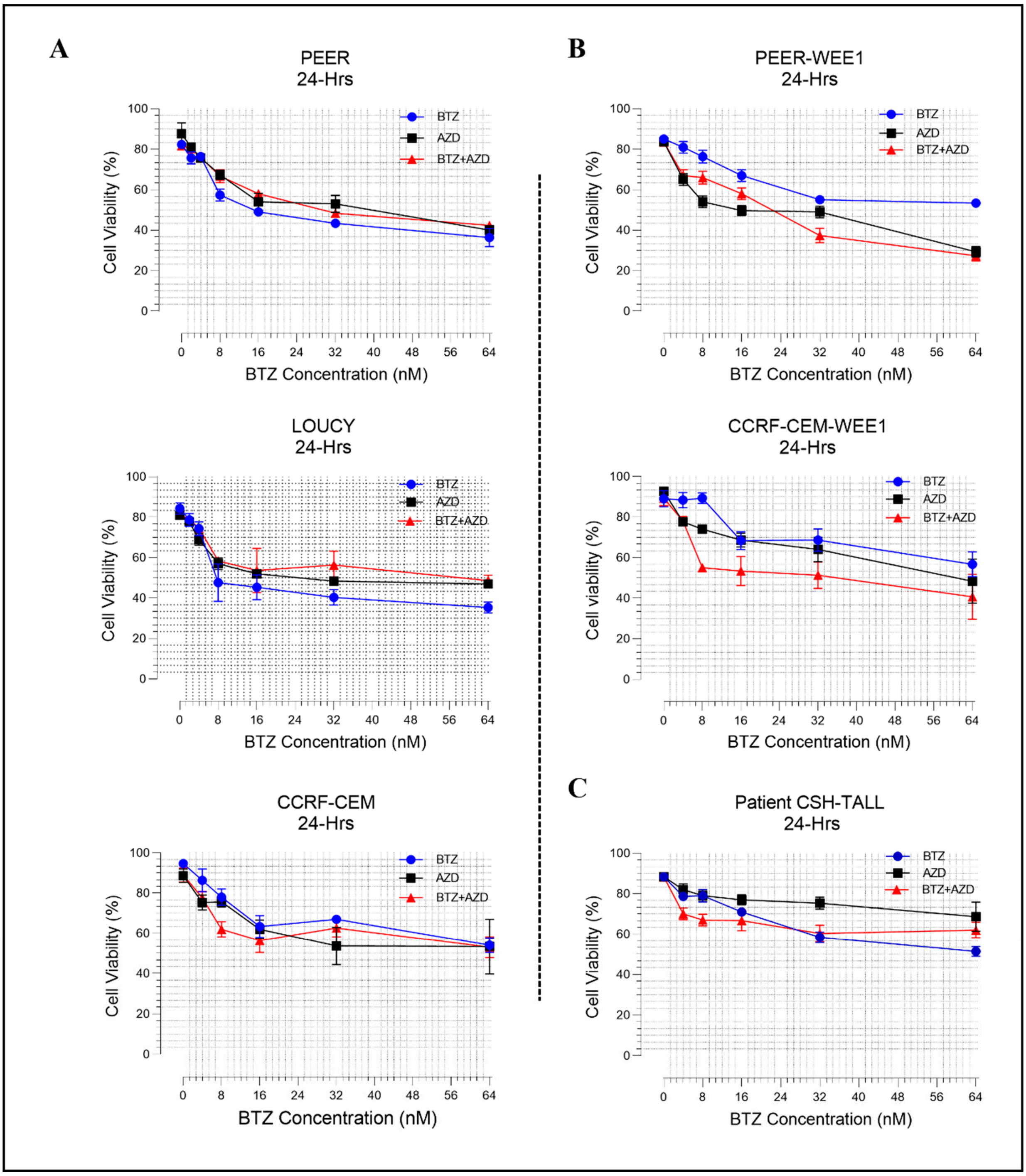

Figure S6. The Effect of Bortezomib (BTZ) Combined with WEE1 Inhibitor (AZD) on T-ALL Cells.

The wild type and WEE1 overexpressing T-ALL cell lines, as well as primary patient's sample (Patient CSH-TALL) were cultured and treated with different concentration of BTZ (Blue) and AZD (Black) alone and in combination (Red) for 24-hours. A concentration gradient of 0 to 64 nM was used for BTZ and 0 to 2560 nM was used for AZD. The cell viability data was recorded. **A:** Corresponds to cell viability data of T-

ALL wild type cell lines. The combination index (CI) was more to 1, which shows antagonistic effect. **B:** Corresponds to cell viability data of WEE1 overexpressing T-ALL cell lines. The combination index (CI) was equal to 1, which shows additive effect. **C:** Corresponds to cell viability data of primary patient's sample which also showed additive effect (CI=1). The experiments were performed independently three times (n=3) and the data presented as mean ± standard error of mean (SEM).

Table S1. The Demographics, Immunophenotype, Cytogenetics and Genomic Information of Patient's Sample.

Patient's Number	TALL01
Patient's Demographics	Male, 31 years-old
Blasts Percentage	>90%
Immunophenotype	Positive: cytoplasmic CD3, CD7, dim CD5 (80%), CD34.<br>Negative: Surface CD3, TCR alpha/beta and TCR gamma/delta, CD1a, CD4, CD8, CD2, CD13, CD33, CD117, MPO.
Cytogenetics	46, XY, add(5)(q13)[4]/46, XY [19]
NGS Targeted Sequencing Findings	KRAS p.G12R, EZH2 p.R34, NOTCH1 p. K1607delinsPAGGE, SF3B1 p.A861delinsDAP

Table S2. Drug Concentrations Individually and in Combination Producing the Same Effect.

T-ALL Cells	Bortezomib (nM)	Adavosertib (nM)	Combine	Combination Index (CI)
PEER	16	640	16 + 640	42.025 > 1
LOUCY	16	640	16 + 640	42.025 > 1
CCRF-CEM	16	640	16 + 640	42.025 > 1
CSH-TALL	14.1176471	80	2 + 10	1 = 1
CCRF-CEM-WEE1	17.6470588	100	3 + 12	1 = 1
PEER-WEE1	17.3913043	200	2 + 14	1 = 1
